# The genome sequence of *Aquilegia vulgaris* L., 1753 (Ranunculales: Ranunculaceae)

**DOI:** 10.12688/wellcomeopenres.26996.1

**Published:** 2026-07-14

**Authors:** Sahr Mian, Maarten J. M. Christenhusz, Royal Botanic Gardens Kew Genome Acquisition Lab, Plant Genome Sizing Collective, Darwin Tree of Life Barcoding collective, Wellcome Sanger Institute Tree of Life Management, Samples and Laboratory team, Wellcome Sanger Institute Scientific Operations: Sequencing Operations, Wellcome Sanger Institute Tree of Life Core Informatics team, Tree of Life Core Informatics collective, Darwin Tree of Life Consortium

**Affiliations:** 1 Royal Botanic Gardens Kew, Richmond, England, UK; 2 Botanical Society of Britain and Ireland, St Albans, Hertfordshire, England, UK

**Keywords:** Aquilegia vulgaris, Columbine, genome sequence, chromosomal, Ranunculales

## Abstract

We present a genome assembly of *Aquilegia vulgaris* (Columbine; Streptophyta; Magnoliopsida; Ranunculales; Ranunculaceae). The assembly consists of two haplotypes with total lengths of 296.10 megabases and 289.22 megabases. Most of haplotype 1 (98.81%) is scaffolded into 7 chromosomal pseudomolecules. Most of haplotype 2 (94.35%) is scaffolded into 7 chromosomal pseudomolecules. The mitochondrial sequences have lengths of 388.22 and 83.91 kilobases and the plastid genome assembly has a length of 161.5 kilobases. Gene annotation of this assembly on Ensembl identified 23 898 protein-coding genes. This assembly was generated as part of the Darwin Tree of Life project, which produces reference genomes for eukaryotic species found in Britain and Ireland.

## Species taxonomy

Eukaryota; Viridiplantae; Streptophyta; Streptophytina; Embryophyta; Tracheophyta; Euphyllophyta; Spermatophyta; Magnoliopsida; Mesangiospermae; Ranunculales; Ranunculaceae; Thalictroideae; *Aquilegia*; *Aquilegia vulgaris* L., 1753 (NCBI:txid3451).

## Background

**Figure 1.**
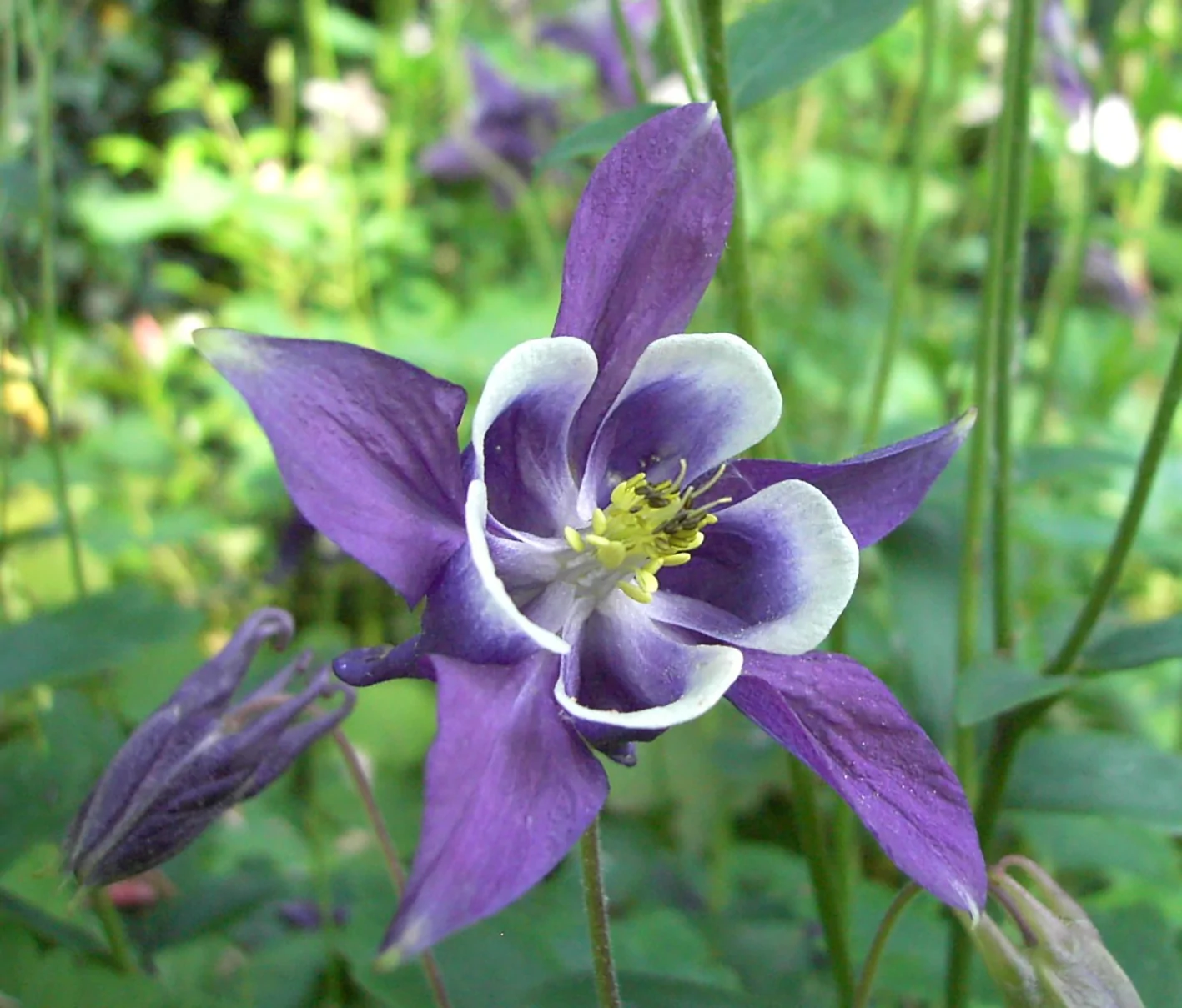
Photograph of *Aquilegia vulgaris* by Arne Nordmann.

*Aquilegia vulgaris* L., commonly known as Columbine ( Figure 1), is a perennial herb of calcareous habitats in Europe ( Fitzgerald & Walker, 2026). In Britain and Ireland, the blue-flowered form has often been treated as native, although its original native range is uncertain. It is associated with chalk and limestone soils and occurs in semi-open habitats, including woodland glades, rides, open scrub, damp grassland, fen, streamsides and scree slopes ( Fitzgerald & Walker, 2026).

The recorded distribution of *A. vulgaris* has expanded substantially since the mid-twentieth century, largely because cultivated plants have escaped from gardens and become naturalised. These naturalised populations occur in a wide range of disturbed habitats, including quarries, roadsides, railway banks, old walls and other ruderal sites ( Fitzgerald & Walker, 2026). This spread has made it difficult to distinguish native from introduced occurrences, and recent distribution records are therefore mapped without native status ( Fitzgerald & Walker, 2026).

The known karyotype of *Aquilegia eximia* is 2 *n* = 14, consistent with the canonical haploid chromosome number of *n* = 7 in *Aquilegia* ( Johns *et al.*, 2026).

We sequenced the genome of Columbine, *Aquilegia vulgaris* as part of the Darwin Tree of Life Project – which aims to generate high-quality reference genomes for all named eukaryotic species in Britain and Ireland to support research, conservation, and the sustainable use of biodiversity.

## Methods

### Sample acquisition, flow cytometry and DNA barcoding

A specimen of *Aquilegia vulgaris* (specimen ID KDTOL10477, ToLID dmAquVulg1) was used for genome sequencing. It was collected from Royal Botanic Gardens Kew, Richmond, Surrey, UK (latitude 51.4823, longitude −0.2881) on 2022-05-06. The specimen was collected by Sahr Mian and identified by Maarten Christenhusz. The dmAquVulg1 specimen was also used for Hi-C and RNA sequencing. The herbarium voucher associated with the sequenced plant is deposited in the herbarium of RBG Kew (K) ( K001400941).

The genome size was estimated by flow cytometry following the ‘one-step’ method outlined in Pellicer *et al*. (2021) and using propidium iodide as the fluorochrome. The General Purpose Buffer (GPB) supplemented with 3% PVP was used for isolation of nuclei ( Loureiro *et al.*, 2007), and the internal calibration standard was *Solanum lycopersicum* L. ‘Stupické polní rané’ with an assumed 1C-value of 968 Mb ( Doležel *et al.*, 2007).

The initial identification was verified by an additional DNA barcoding process according to the framework developed by Twyford *et al*. (2024). Part of the plant specimen was preserved in silica gel desiccant ( Chase & Hills, 1991). DNA extracted from the dried plant was amplified by PCR for standard barcode markers, with the amplicons sequenced and compared to public sequence databases including GenBank and the Barcode of Life Database (BOLD) ( Ratnasingham & Hebert, 2007). Following whole genome sequence generation, the relevant DNA barcode region was also used alongside the initial barcoding data for sample tracking at the WSI ( Twyford *et al.*, 2024). The standard operating procedures for Darwin Tree of Life barcoding are available on protocols.io.

### Nucleic acid extraction

Protocols for high molecular weight (HMW) DNA extraction developed at the Wellcome Sanger Institute (WSI) Tree of Life Core Laboratory are available on protocols.io ( Howard *et al.*, 2025). The dmAquVulg1 sample was weighed and triaged to determine the appropriate extraction protocol. For HMW DNA extraction, 51.0 mg of leaf tissue was used. The tissue was homogenised by cryogenic disruption using the Covaris cryoPREP® Automated Dry Pulverizer. HMW DNA was extracted using the Automated Plant MagAttract v3 protocol. We used centrifuge-mediated fragmentation to produce DNA fragments in the 8–10 kb range, following the Covaris g-TUBE protocol for ultra-low input (ULI). Sheared DNA was purified by automated SPRI (solid-phase reversible immobilisation), using AMPure PB beads (Pacific Biosciences) and the Thermo Fisher KingFisher™ Apex to eliminate shorter fragments and concentrate the DNA.

The concentration of the sheared and purified DNA was assessed using a Nanodrop spectrophotometer and Qubit Fluorometer using the Qubit dsDNA High Sensitivity Assay kit. Fragment size distribution was evaluated by running the sample on the FemtoPulse system. For this sample, the final post-shearing DNA had a Qubit concentration of 2.22 ng/μL, with a fragment size of 8.6 kb.

RNA was extracted from leaf tissue of dmAquVulg1 in the Tree of Life Laboratory at the WSI using the RNA Extraction: Automated MagMax™ *mir*Vana protocol. The RNA concentration was assessed using a Nanodrop spectrophotometer and a Qubit Fluorometer using the Qubit RNA Broad-Range Assay kit. Analysis of the integrity of the RNA was done using the Agilent RNA 6000 Pico Kit and Eukaryotic Total RNA assay.

### PacBio HiFi library preparation and sequencing

Library preparation and sequencing were performed at the WSI Scientific Operations core. Prior to library preparation, the DNA was fragmented to ~10 kb. Ultra-low-input (ULI) libraries were prepared using the PacBio SMRTbell® Express Template Prep Kit 2.0 and gDNA Sample Amplification Kit. Samples were normalised to 20 ng DNA. Single-strand overhang removal, DNA damage repair, and end-repair/A-tailing were performed according to the manufacturer’s instructions, followed by adapter ligation. A 0.85× pre-PCR clean-up was carried out with Promega ProNex beads.

The DNA was evenly divided into two aliquots for dual PCR (reactions A and B), both following the manufacturer’s protocol. A 0.85× post-PCR clean-up was performed with ProNex beads. DNA concentration was measured using a Qubit Fluorometer v4.0 (Thermo Fisher Scientific) with the Qubit HS Assay Kit, and fragment size was assessed on an Agilent Femto Pulse Automated Pulsed Field CE Instrument (Agilent Technologies) using the gDNA 55 kb BAC analysis kit. PCR reactions A and B were then pooled, ensuring a total mass of ≥500 ng in 47.4 μl.

The pooled sample underwent another round of DNA damage repair, end-repair/A-tailing, and hairpin adapter ligation. A 1× clean-up was performed with ProNex beads, followed by DNA quantification using the Qubit and fragment size analysis using the Agilent Femto Pulse. Size selection was performed on the Sage Sciences PippinHT system, with target fragment size determined by Femto Pulse analysis (typically 4–9 kb). Size-selected libraries were cleaned with 1.0× ProNex beads and normalised to 2 nM before sequencing.

The sample was sequenced using the Sequel IIe system (Pacific Biosciences, California, USA). The concentration of the library loaded onto the Sequel IIe was in the range 40–135 pM. SMRT Link software, a PacBio web-based workflow manager, was used to set up and monitor the run, and to perform primary and secondary analysis of the data upon completion.

### Hi-C

***Sample preparation and crosslinking***

Hi-C data were generated from the leaf tissue of dmAquVulg1 using the Arima-HiC v2 kit (Arima Genomics). Tissue was finely ground using the Covaris cryoPREP Dry Pulverizer (Covaris), and then subjected to nuclei isolation. Nuclei were isolated using a modified protocol based on the Qiagen QProteome Cell Compartment Kit (Qiagen), in which only the Lysis and CE2 buffers were used, with QIAshredder spin columns. After isolation, nuclei were fixed using formaldehyde to a final concentration of 2% to crosslink the DNA. The crosslinked DNA was then digested and biotinylated according to the manufacturer’s instructions. A clean-up step was performed with SPRIselect beads before library preparation. DNA concentration was quantified using the Qubit Fluorometer v4.0 (Thermo Fisher Scientific) and the Qubit HS Assay Kit, following the manufacturer’s instructions.

***Hi-C library preparation and sequencing***

Biotinylated DNA constructs were fragmented using a Covaris E220 sonicator and size selected to 400–600 bp using SPRISelect beads. DNA was enriched with Arima-HiC v2 kit Enrichment beads. End repair, A-tailing, and adapter ligation were carried out with the NEBNext Ultra II DNA Library Prep Kit (New England Biolabs), following a modified protocol where library preparation occurs while DNA remains bound to the Enrichment beads. Library amplification was performed using KAPA HiFi HotStart mix and a custom Unique Dual Index (UDI) barcode set (Integrated DNA Technologies). Depending on sample concentration and biotinylation percentage determined at the crosslinking stage, libraries were amplified with 10–16 PCR cycles. Post-PCR clean-up was performed with SPRISelect beads. Libraries were quantified using the AccuClear Ultra High Sensitivity dsDNA Standards Assay Kit (Biotium) and a FLUOstar Omega plate reader (BMG Labtech).

Prior to sequencing, libraries were normalised to 10 ng/μL. Normalised libraries were quantified again to create equimolar and/or weighted 2.8 nM pools. Pool concentrations were checked using the Agilent 4200 TapeStation (Agilent) with High Sensitivity D500 reagents before sequencing. Sequencing was performed using paired-end 150 bp reads on the Illumina NovaSeq 6000.

### RNA library preparation and sequencing

Libraries were prepared using the NEBNext® Ultra II Directional RNA Library Prep Kit for Illumina (New England Biolabs), following the manufacturer’s instructions. Poly(A) mRNA was isolated from 100 ng of total RNA using oligo (dT) beads, converted to cDNA, and uniquely indexed; 14 PCR cycles were performed. Libraries were prepared with an intended fragment size of 100–300 bp. Libraries were quantified, normalised, and pooled equimolarly to a final concentration of 2.8 nM before dilution to 150 pM for loading. Sequencing was carried out on the Illumina NovaSeq 6000, generating paired-end reads.

### Genome assembly

Prior to assembly of the PacBio HiFi reads, a database of *k*-mer counts ( *k* = 31) was generated from the filtered reads using FastK. GenomeScope2 ( Ranallo-Benavidez *et al.*, 2020) was used to analyse the *k*-mer frequency distributions, providing estimates of genome size, heterozygosity, and repeat content.

The HiFi reads were assembled using Hifiasm in Hi-C phasing mode ( Cheng *et al.*, 2021, 2022), producing two haplotypes. Hi-C reads ( Rao *et al.*, 2014) were mapped to the primary contigs using bwa-mem2 ( Vasimuddin *et al.*, 2019). Contigs were further scaffolded with Hi-C data in YaHS ( Zhou *et al.*, 2023), using the --break option for handling potential misassemblies. The scaffolded assemblies were evaluated using Gfastats ( Formenti *et al.*, 2022), BUSCO ( Manni *et al.*, 2021) and MerquryFK ( Rhie *et al.*, 2020). The organelle genomes were assembled using OATK ( Zhou *et al.*, 2025).

### Assembly curation

The assembly was decontaminated using the Assembly Screen for Cobionts and Contaminants ( ASCC) pipeline. TreeVal was used to generate the flat files and maps for use in curation. Manual curation was conducted primarily in PretextView and HiGlass ( Kerpedjiev *et al.*, 2018). Scaffolds were visually inspected and corrected as described by Howe *et al*. (2021). Manual corrections included 48 breaks and 178 joins. This reduced the scaffold count by 26.7%, increased the scaffold N50 by 9.5%, and increased the total assembly length by 1.0%. The curation process is described at https://gitlab.com/wtsi-grit/rapid-curation . PretextSnapshot was used to generate a Hi-C contact map of the final assembly.

### Assembly quality assessment

The MerquryFK tool ( Rhie *et al.*, 2020) was run in a Singularity container ( Kurtzer *et al.*, 2017) to evaluate *k*-mer completeness and assembly quality for both haplotypes using the *k*-mer database ( *k* = 31) computed prior to genome assembly. The analysis outputs included assembly QV scores and completeness statistics.

The genome was analysed using the BlobToolKit pipeline, a Nextflow implementation of the earlier Snakemake version ( Challis *et al.*, 2020). The pipeline aligns PacBio reads using minimap2 ( Li, 2018) and SAMtools ( Danecek *et al.*, 2021) to generate coverage tracks. It runs BUSCO ( Manni *et al.*, 2021) using lineages identified by querying NCBI datasets ( O’Leary *et al.*, 2024). For the three domain-level lineages, BUSCO genes are aligned to the UniProt Reference Proteomes database ( Bateman *et al.*, 2023) using DIAMOND blastp ( Buchfink *et al.*, 2021). The genome is divided into chunks based on the density of BUSCO genes from the closest taxonomic lineage, and each chunk is aligned to the UniProt Reference Proteomes database with DIAMOND blastx. Sequences without hits are chunked using seqtk and aligned to the NT database with blastn ( Altschul *et al.*, 1990). The BlobToolKit suite consolidates all outputs into a blobdir for visualisation. The BlobToolKit pipeline was developed using nf-core tooling ( Ewels *et al.*, 2020) and MultiQC ( Ewels *et al.*, 2016), with containerisation through Docker ( Merkel, 2014) and Singularity ( Kurtzer *et al.*, 2017).

## Genome sequence report

### Sequence data

**Figure 2.**
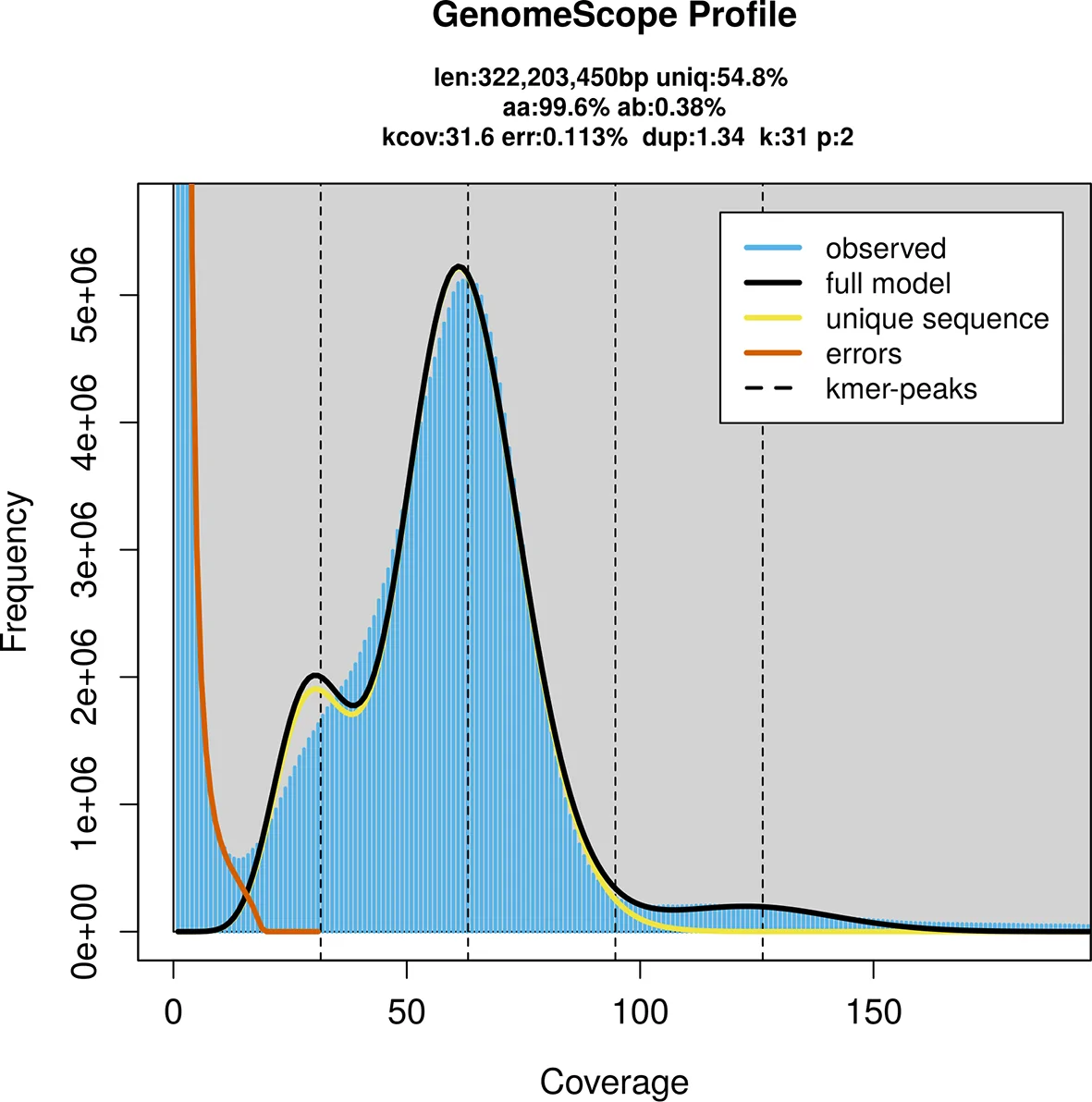
Frequency distribution of *k*-mers generated using GenomeScope2. The plot shows observed and modelled *k*-mer spectra, providing estimates of genome size, heterozygosity, and repeat content based on unassembled sequencing reads.

The genome of a specimen of *Aquilegia vulgaris* was sequenced using Pacific Biosciences single-molecule HiFi long reads, generating 21.47 Gb (gigabases) from 2.62 million reads, which were used to assemble the genome. GenomeScope2.0 analysis estimated the haploid genome size at 322.77 Mb, with a heterozygosity of 0.38% and repeat content of 45.19% ( Figure 2). Using flow cytometry, the genome size (1C-value) of the sample was estimated to be 0.41 pg, equivalent to 400.00 Mb. These estimates guided expectations for the assembly. Based on the estimated genome size, the sequencing data provided approximately 63× coverage. Hi-C sequencing produced 106.91 Gb from 354.01 million reads, which were used to scaffold the assembly. RNA sequencing data were also generated and are available in public sequence repositories. Table 1 summarises the specimen and sequencing details.

**Table 1.** Specimen and sequencing data for *Aquilegia vulgaris* (BioProject PRJEB77580).

Platform	PacBio HiFi	Hi-C	RNA-seq
**ToLID**	dmAquVulg1	dmAquVulg1	dmAquVulg1
**Specimen ID**	KDTOL10477	KDTOL10477	KDTOL10477
**BioSample (source individual)**	SAMEA14449597	SAMEA14449597	SAMEA14449597
**BioSample (tissue)**	SAMEA14449685	SAMEA14449686	SAMEA14449685
**Tissue**	leaf	leaf	leaf
**Instrument**	Sequel IIe	Illumina NovaSeq 6000	Illumina NovaSeq 6000
**Run accessions**	ERR13382505	ERR13363473	ERR13363474
**Read count total**	2.62 million reads	354.01 million read pairs	32.16 million read pairs
**Base count total**	21.47 Gb	106.91 Gb	9.71 Gb

### Assembly statistics

The genome was assembled into two haplotypes using Hi-C phasing. Haplotype 1 was curated to chromosome level, while haplotype 2 was assembled to scaffold level. The final assembly has a total length of 296.10 Mb in 134 scaffolds, with 433 gaps, and a scaffold N50 of 42.72 Mb ( Table 2).

**Table 2.** Genome assembly statistics for *Aquilegia vulgaris.*

Genome assembly	Haplotype 1	Haplotype 2
**Assembly name**	dmAquVulg1.hap1.1	dmAquVulg1.hap2.1
**Assembly accession**	GCA_964291885.1	GCA_964291825.1
**Assembly level**	chromosome	chromosome
**Span (Mb)**	296.10	289.22
**Number of chromosomes**	7	7
**Number of contigs**	567	854
**Contig N50**	1.17 Mb	1.17 Mb
**Number of scaffolds**	134	483
**Scaffold N50**	42.72 Mb	37.74 Mb
**Longest scaffold length (Mb)**	51.47	46.91
**Organelles**	Mitochondrial genomes: 388.22 and 83.91 kb; Plastid genome: 161.50 kb	-

The scaffold count follows the NCBI assembly statistics and excludes organelle assemblies, which are reported separately where present.

**Figure 3.**
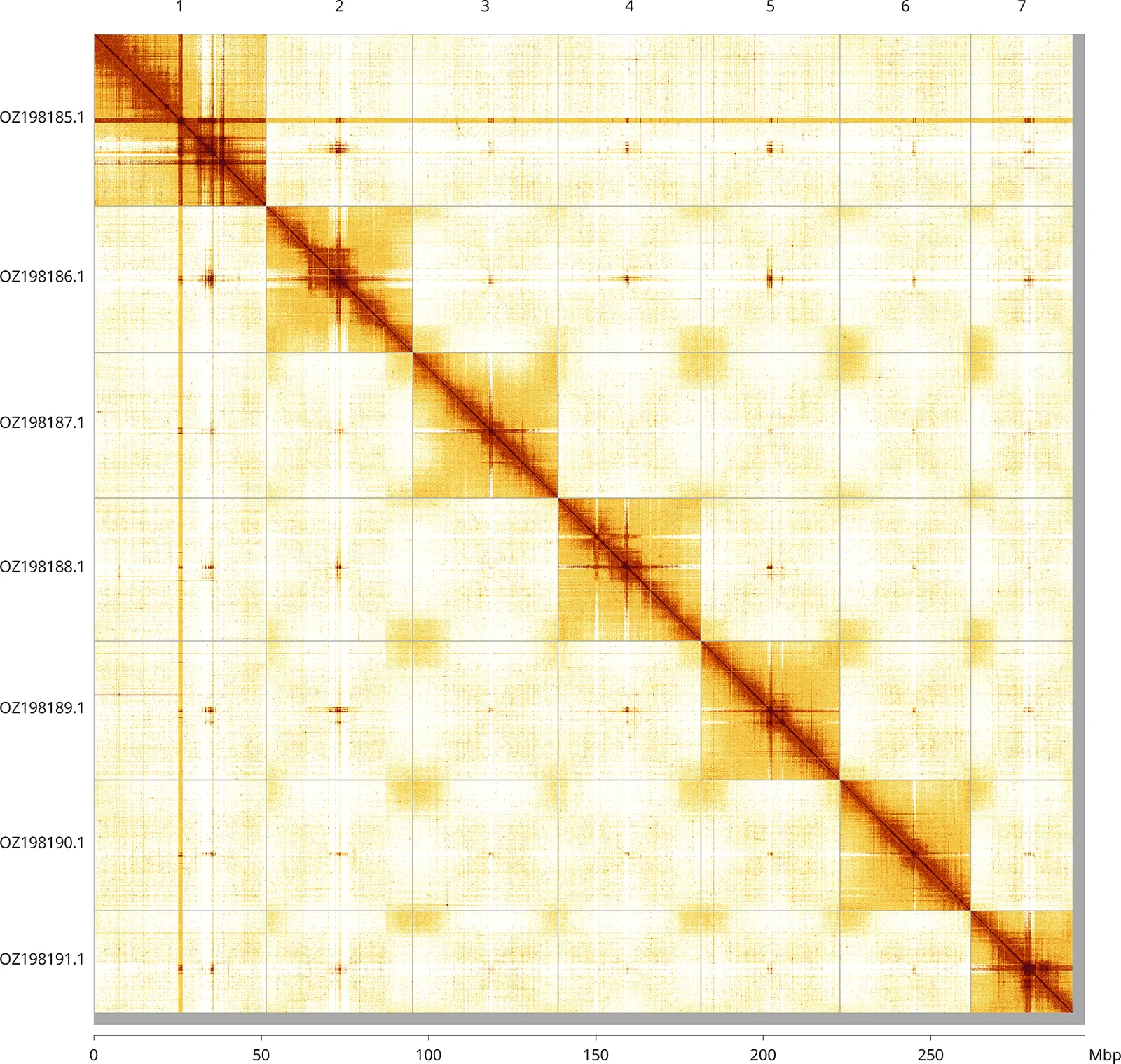
Hi-C contact map of the *Aquilegia vulgaris* genome assembly. Assembled chromosomes are shown in order of size and labelled along the axes, with a megabase scale shown below. The plot was generated using PretextSnapshot.

Most of the assembly sequence (98.81%) was assigned to 7 chromosomal-level scaffolds. These chromosome-level scaffolds, confirmed by Hi-C data, are named according to size ( Figure 3; Table 3). During curation, we observed that the order and orientation of the contigs on chromosome 1 (25.3–26.6 Mbp) are uncertain.

**Table 3.** Chromosomal pseudomolecules in both haplotypes of the genome assembly of *Aquilegia vulgaris*, dmAquVulg1.

Haplotype 1				Haplotype 2			
INSDC accession	Name	Length (Mb)	GC%	INSDC accession	Name	Length (Mb)	GC%
OZ198185.1	1	51.47	38	OZ198195.1	1	46.91	37.50
OZ198186.1	2	43.80	37.50	OZ198199.1	2	37.69	37
OZ198187.1	3	43.44	37	OZ198196.1	3	43.65	37
OZ198188.1	4	42.72	37	OZ198197.1	4	40.77	37
OZ198189.1	5	41.52	37	OZ198198.1	5	37.74	36.50
OZ198190.1	6	39.02	36.50	OZ198200.1	6	37.38	36.50
OZ198191.1	7	30.60	37	OZ198201.1	7	28.75	37

Two mitochondrial genomes (lengths 388.22 kb [OZ198192.1] and 83.91 kb [OZ198193.1]) and the plastid genome (length 161.5 kb, OZ198194.1) were also assembled. These sequences are included as contigs in the multifasta file of the genome submission and as standalone records.

### Assembly quality metrics

**Figure 4.**
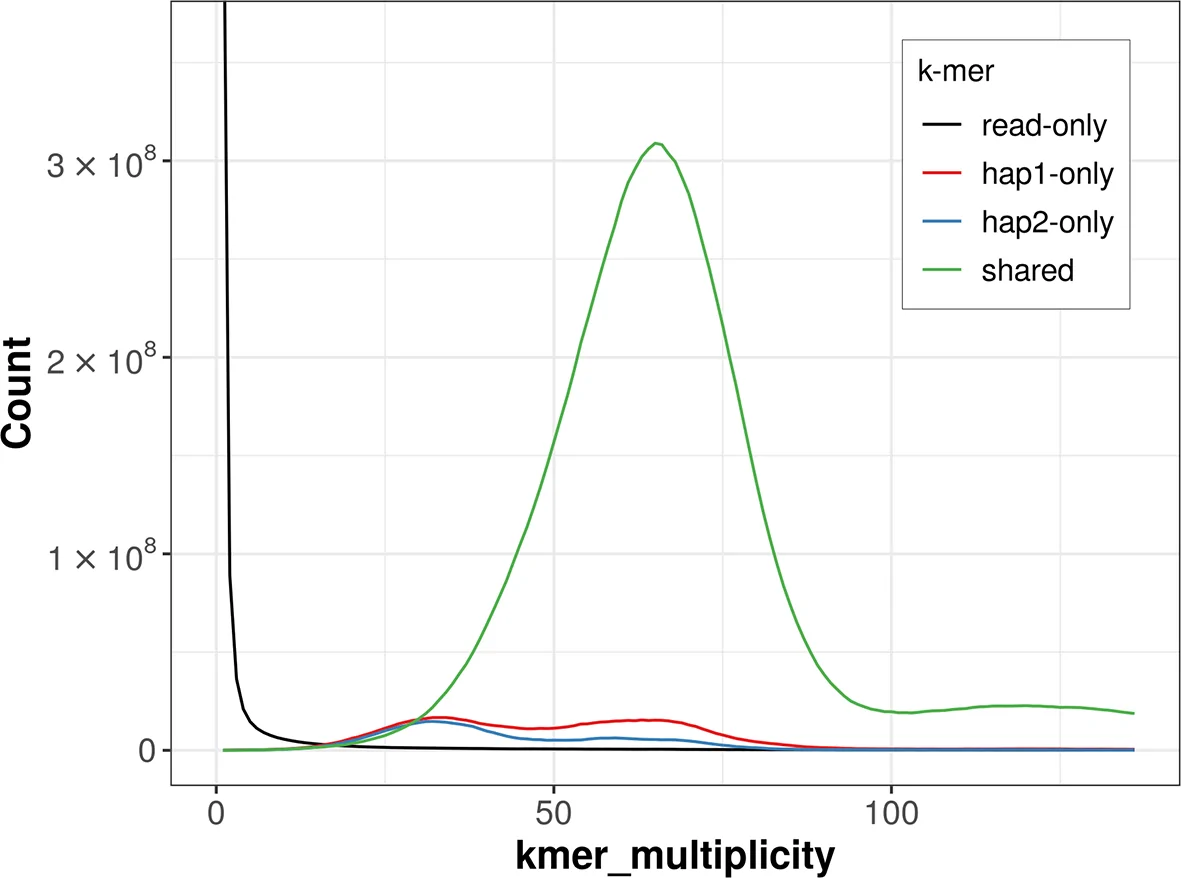
Evaluation of *k*-mer completeness using MerquryFK. This plot illustrates the recovery of *k*-mers from the original read data in the final assemblies. The horizontal axis represents *k*-mer multiplicity, and the vertical axis shows the number of *k*-mers. The black curve represents *k*-mers that appear in the reads but are not assembled. The green curve corresponds to *k*-mers shared by both haplotypes, and the red and blue curves show *k*-mers found only in one of the haplotypes.

For haplotype 1, the estimated QV is 61.6, and for haplotype 2, 59.4. When the two haplotypes are combined, the assembly achieves an estimated QV of 60.4. The *k*-mer completeness is 92.81% for haplotype 1, 89.70% for haplotype 2, and 98.70% for the combined haplotypes ( Figure 4).

**Figure 5.**
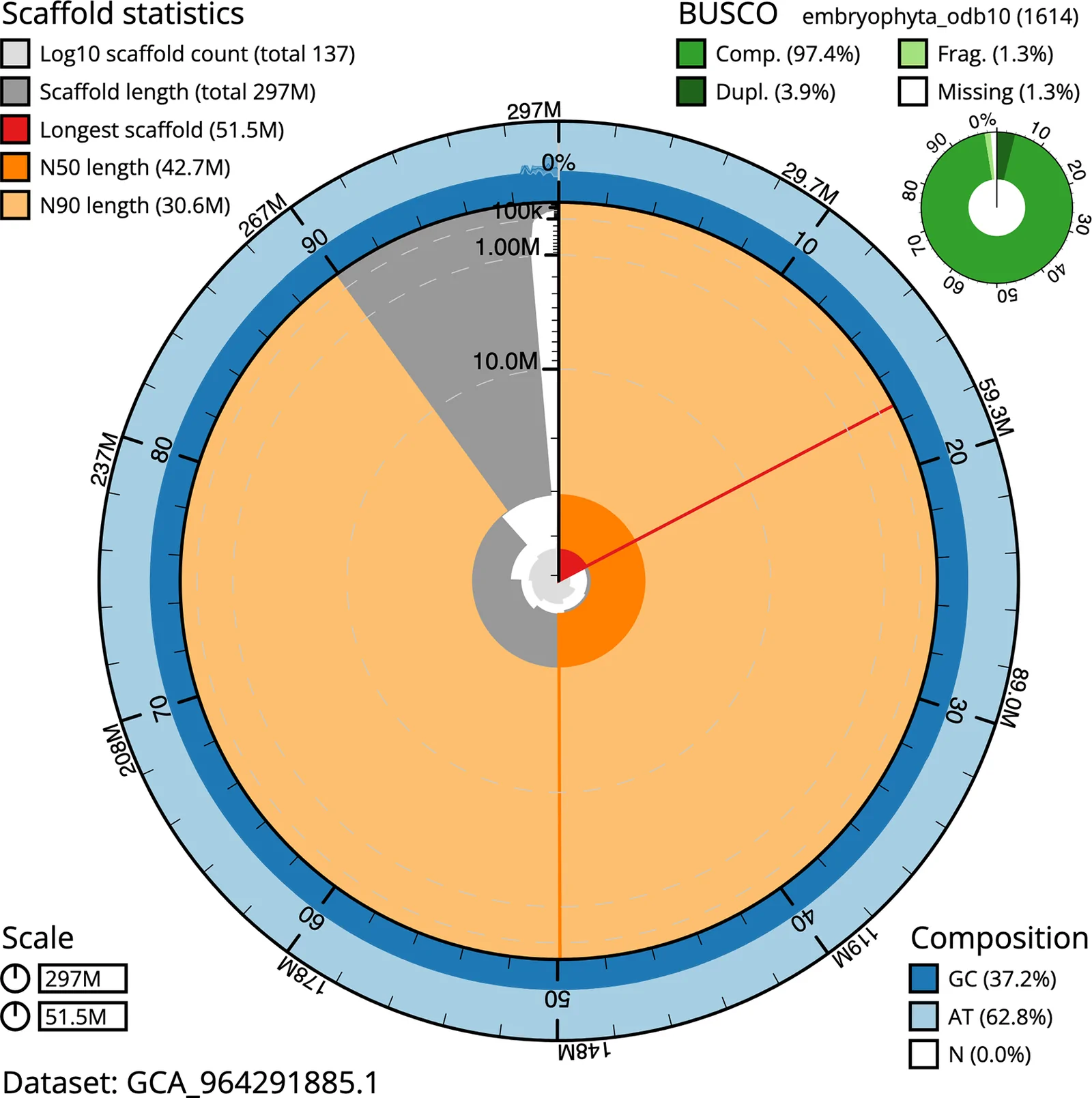
Assembly metrics for dmAquVulg1.hap1.1. The BlobToolKit snail plot provides an overview of assembly metrics and BUSCO gene completeness. The circumference represents the length of the whole genome sequence, and the main plot is divided into 1 000 bins around the circumference. The outermost blue tracks display the distribution of GC, AT, and N percentages across the bins. Scaffolds are arranged clockwise from longest to shortest and are depicted in dark grey. The longest scaffold is indicated by the red arc, and the deeper orange and pale orange arcs represent the N50 and N90 lengths. A light grey spiral at the centre shows the cumulative scaffold count on a logarithmic scale. A summary of complete, fragmented, duplicated, and missing BUSCO genes in the set is presented at the top right. An interactive version of this figure can be accessed on the BlobToolKit viewer.

**Figure 6.**
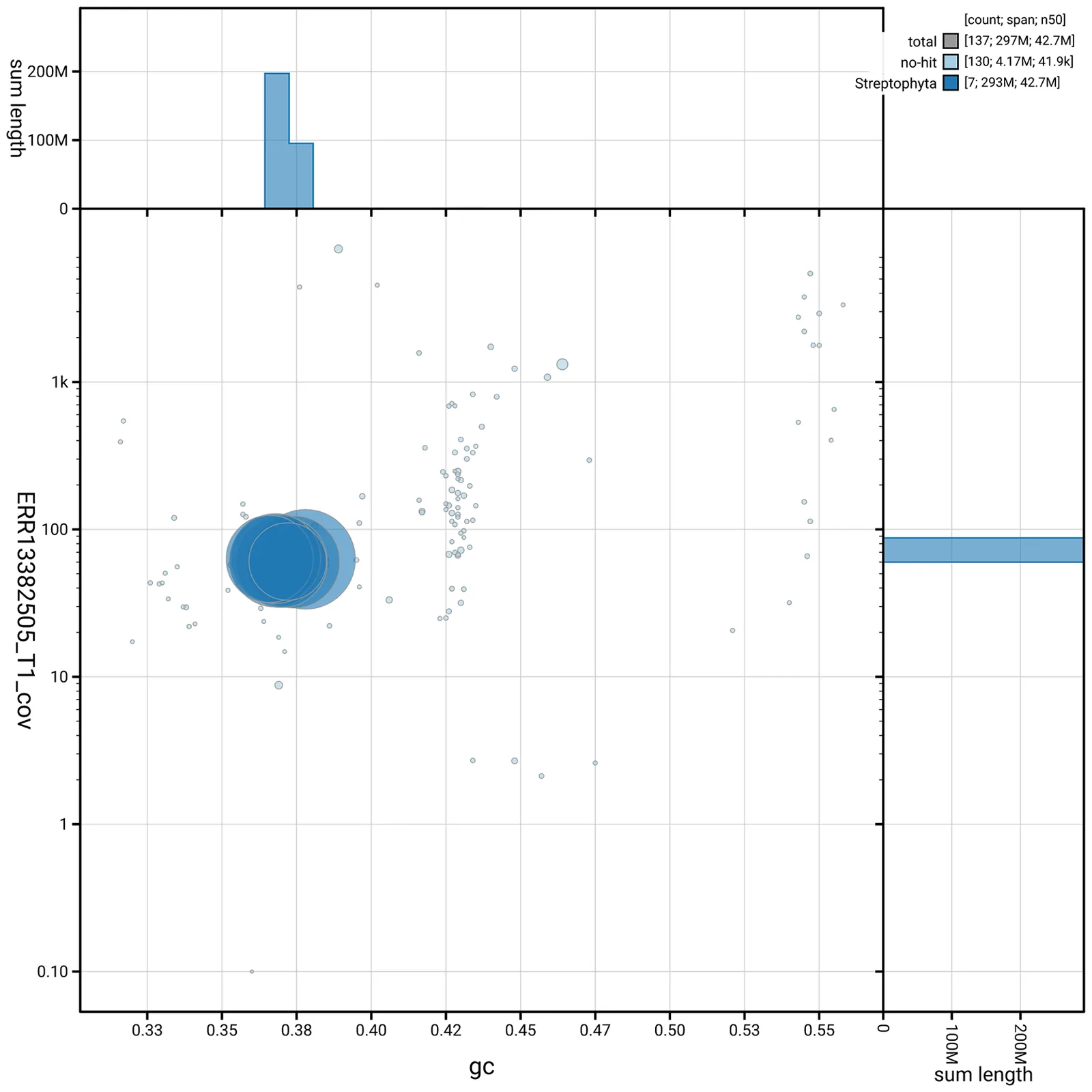
BlobToolKit blob plot for dmAquVulg1.hap1.1. The plot shows base coverage (vertical axis) and GC content (horizontal axis). The circles represent scaffolds, with the size proportional to scaffold length and the colour representing phylum membership. The histograms along the axes display the total length of sequences distributed across different levels of coverage and GC content. An interactive version of this figure is available on the BlobToolKit viewer.

BUSCO analysis using the embryophyta_odb10 reference set ( *n* = 1 614) identified 97.4% of the expected gene set (single = 93.5%, duplicated = 3.9%) for haplotype 1. The snail plot in Figure 5 summarises the scaffold length distribution and other assembly statistics for haplotype 1. The blob plot in Figure 6 shows the distribution of scaffolds by GC proportion and coverage for haplotype 1.

Table 4 lists the assembly metric benchmarks adapted from Rhie *et al.* (2021) and the Earth BioGenome Project Report on Assembly Standards January 2026. The EBP metric calculated for the haplotype 1 is **6.C.Q61**, meeting the recommended 6.C.Q40 reference standard.

**Table 4.** Earth Biogenome Project summary metrics for the *Aquilegia vulgaris* assembly.

Measure	Value	Benchmark
EBP summary (haplotype 1)	6.C.Q61	6.C.Q40
Contig N50 length	1.17 Mb	≥ 1 Mb
Scaffold N50 length	42.72 Mb	= chromosome N50
Consensus quality (QV)	Haplotype 1: 61.6; haplotype 2: 59.4; combined: 60.4	≥ 40
*k*-mer completeness	Haplotype 1: 92.81%; Haplotype 2: 89.70%; combined: 98.70%	≥ 95%
BUSCO	Haplotype 1: C:97.4% [S:93.5%, D:3.9%], F:1.3%, M:1.3%, n:1 614; Haplotype 2: C:94.5% [S:90.7%, D:3.8%], F:1.5%, M:4.0%, n:1 614	S > 90%; D < 5%
Percentage of assembly assigned to chromosomes	98.81%	≥ 90%

*Notes*: The EBP summary uses log10(Contig N50); chromosome-level (C) or log10(Scaffold N50); Q (Merqury QV). BUSCO: C = complete; S = single-copy; D = duplicated; F = fragmented; M = missing; n = orthologues

**Table 5.** Software versions and sources used for *Aquilegia vulgaris.*

Software	Version	Source
BLAST	2.14.0	ftp://ftp.ncbi.nlm.nih.gov/blast/executables/blast+/
BlobToolKit	4.3.9	https://github.com/blobtoolkit/blobtoolkit
BUSCO	5.5.0; 5.7.1	https://gitlab.com/ezlab/busco
bwa-mem2	2.2.1	https://github.com/bwa-mem2/bwa-mem2
DIAMOND	2.1.8	https://github.com/bbuchfink/diamond
fasta_windows	0.2.4	https://github.com/tolkit/fasta_windows
FastK	1.1	https://github.com/thegenemyers/FASTK
GenomeScope2.0	2.0.1	https://github.com/tbenavi1/genomescope2.0
Gfastats	1.3.6	https://github.com/vgl-hub/gfastats
Hifiasm	0.19.8-r603	https://github.com/chhylp123/hifiasm
HiGlass	1.13.4	https://github.com/higlass/higlass
MerquryFK	1.1.0-c1	https://github.com/thegenemyers/MERQURY.FK
Minimap2	2.24-r1122	https://github.com/lh3/minimap2
Oatk	0.9	https://github.com/c-zhou/oatk
MultiQC	1.14; 1.17 and 1.18	https://github.com/MultiQC/MultiQC
Nextflow	23.10.0; 23.10.1	https://github.com/nextflow-io/nextflow
PretextSnapshot	0.0.4	https://github.com/sanger-tol/PretextSnapshot
PretextView	1.0.3	https://github.com/sanger-tol/PretextView
samtools	1.17; 1.18; 1.19.2; 1.21; 1.6	https://github.com/samtools/samtools
sanger-tol/ascc	0.1.0	https://github.com/sanger-tol/ascc
sanger-tol/blobtoolkit	0.6.0	https://github.com/sanger-tol/blobtoolkit
sanger-tol/curationpretext	1.4.2	https://github.com/sanger-tol/curationpretext
Seqtk	1.4-r122	https://github.com/lh3/seqtk
Singularity	3.9.0	https://github.com/sylabs/singularity
TreeVal	1.1.1	https://github.com/sanger-tol/treeval
YaHS	1.2a.2	https://github.com/c-zhou/yahs

### Genome annotation report

The *Aquilegia vulgaris* genome assembly (GCA_964291885.1) was annotated by Ensembl at the European Bioinformatics Institute (EBI). This annotation includes 48 742 transcribed mRNAs from 23 898 protein-coding and 11 547 non-coding genes. The average transcript length is 3 368.60 bp, with an average of 1.38 coding transcripts per gene and 4.84 exons per transcript. For further information about the annotation, please refer to the annotation page on Ensembl.

## Author information

• Members of the Royal Botanic Gardens Kew Genome Acquisition Lab
• Members of the Plant Genome Sizing Collective
• Members of the Darwin Tree of Life Barcoding collective
• Members of the Wellcome Sanger Institute Tree of Life Management, Samples and Laboratory team
• Members of Wellcome Sanger Institute Scientific Operations – Sequencing Operations
• Members of the Wellcome Sanger Institute Tree of Life Core Informatics team
• Members of the Tree of Life Core Informatics collective
• Members of the Darwin Tree of Life Consortium

## Wellcome Sanger Institute – Legal and governance

The materials that have contributed to this genome note have been supplied by a Darwin Tree of Life Partner. The submission of materials by a Darwin Tree of Life Partner is subject to the **‘Darwin Tree of Life Project Sampling Code of Practice’**, which can be found in full on the Darwin Tree of Life website. By agreeing with and signing up to the Sampling Code of Practice, the Darwin Tree of Life Partner agrees they will meet the legal and ethical requirements and standards set out within this document in respect of all samples acquired for, and supplied to, the Darwin Tree of Life Project. Further, the Wellcome Sanger Institute employs a process whereby due diligence is carried out proportionate to the nature of the materials themselves, and the circumstances under which they have been/are to be collected and provided for use. The purpose of this is to address and mitigate any potential legal and/or ethical implications of receipt and use of the materials as part of the research project, and to ensure that in doing so we align with best practice wherever possible. The overarching areas of consideration are:

• Ethical review of provenance and sourcing of the material
• Legality of collection, transfer and use (national and international)

Each transfer of samples is further undertaken according to a Research Collaboration Agreement or Material Transfer Agreement entered into by the Darwin Tree of Life Partner, Genome Research Limited (operating as the Wellcome Sanger Institute), and in some circumstances, other Darwin Tree of Life collaborators.

## Data Availability

European Nucleotide Archive: Aquilegia vulgaris. Accession number PRJEB77580; https://identifiers.org/ena.embl/PRJEB77580. The genome sequence is released openly for reuse. The *Aquilegia vulgaris* genome sequencing initiative is part of the Darwin Tree of Life Project (PRJEB40665) and the Sanger Institute Tree of Life Programme (PRJEB43745). All raw sequence data and the assembly have been deposited in INSDC databases. Raw data and assembly accession identifiers are reported in Tables 1 and 2. Pipelines used for genome assembly at the WSI Tree of Life are available at https://pipelines.tol.sanger.ac.uk/pipelines. Table 5 lists software versions used in this study.
