# Supplementary material for: The genome sequence of Aquilegia vulgaris L., 1753 (Ranunculales: Ranunculaceae)
Source: Wellcome Open Res. 2026 Jul 14;11:440. [Version 1] doi: 10.12688/wellcomeopenres.26996.1 (PMC13570118; doi:10.12688/wellcomeopenres.26996.1)
Supplement: Peer review history

# Reviewer report: Reviewer response for version 1

**DOI:** 10.21956/wellcomeopenres.29717.r165960

**Published:** 2026-09-11

**Authors:** Zhigui Bao

**Affiliations:** 1 Max Planck Institute for Biology Tübingen, Tübingen, Germany

Mian et al. assembled a chromosome-level reference genome for *Aquilegia vulgaris*. Overall, the genome assembly appears to be of high quality, with good contiguity and chromosome-scale scaffolding. The assembly and Hi-C results are generally convincing, and the resource should be valuable for future comparative and evolutionary genomic studies in *Aquilegia*. However, several aspects of the assembly validation, chromosome structure, and genome annotation would benefit from additional clarification or analysis.

1. **Telomere and centromere annotation**
  Were telomeric sequences successfully assembled at both ends of all seven chromosomes? Given the high contiguity of the assembly, it would be useful to explicitly report the presence or absence of telomeric repeats at each chromosome end. 
  In addition, the Hi-C contact map appears to show strong interaction patterns associated with putative centromeric regions across the chromosomes. It would be helpful to characterize these regions more explicitly. For example, the authors could use a tandem-repeat annotation tool such as TRASH, together with repeat abundance and chromosomal distribution, to identify candidate centromeric satellite repeats and annotate the approximate centromere positions.
2. **Uncertainty in the Chr1 assembly**
  The alternative assemblies/curations shown for Chr1 appear to differ mainly because of one relatively small contig, while another region seems to show an inversion-like configuration. Could the authors clarify the evidence supporting the final Chr1 structure?
  In particular, is it possible that the apparent inversion represents a heterozygous structural variant between the two haplotypes rather than an assembly error? If the final reference was generated by curating a single haplotype, it would be useful to determine whether this structural difference reflects true haplotype-specific variation. Long-read alignments, haplotype-resolved assembly comparison, or additional Hi-C evidence around the inversion breakpoints could help distinguish between these possibilities.
3. **Genome annotation quality**
  The description of the genome annotation is currently rather brief, and the reported value of approximately 43% “homology coverage” is difficult to interpret without further explanation. Please clarify how this metric was calculated, what reference protein or transcript datasets were used, and what biological meaning should be attributed to this percentage.
  More standard annotation-quality metrics would make the annotation easier to evaluate and compare with other plant genomes. In particular, I suggest reporting BUSCO completeness for the predicted protein set, in addition to statistics such as the number of protein-coding genes, average gene/transcript length, exon number per gene, proportion of genes supported by transcript or protein evidence, and the fraction of predicted proteins with functional annotations.
4. **Consistency with related *Aquilegia* genomes**
  Because reference genomes are already available for other *Aquilegia* species, a chromosome-level synteny comparison could provide an additional independent assessment of the assembly. Such a comparison would be particularly informative for Chr1 and could help determine whether the observed structural differences are specific to *A. vulgaris*, represent heterozygous variation, or potentially result from assembly/curation decisions.

Overall, the genome assembly appears to be of good quality, and these points are mainly intended to improve the characterization and validation of an already strong genomic resource.

Are sufficient details of methods and materials provided to allow replication by others?

Yes

Is the rationale for creating the dataset(s) clearly described?

Yes

Are the datasets clearly presented in a useable and accessible format?

Yes

Are the protocols appropriate and is the work technically sound?

Yes

Reviewer Expertise:

computational biology

I confirm that I have read this submission and believe that I have an appropriate level of expertise to confirm that it is of an acceptable scientific standard.

# Reviewer report: Reviewer response for version 1

**DOI:** 10.21956/wellcomeopenres.29717.r165964

**Published:** 2026-09-10

**Authors:** Alexandros Bousios

**Affiliations:** 1 University of Sussex, Brighton, UK

This appears like a standard Genome Note that comes from DToL genomes, it all looks good to me. One point for the authors to check and try to improve is the orientation of contigs in Chr1: the HiC map looks like it is capturing the connection between the centromeres of every chromosomes (the red dots), but Chr1 has two points of contact. Can this be sorted?

 Also why "Haplotype 1 was curated to chromosome level, while haplotype 2 was assembled to scaffold level" when Table 2 (and the metrics for both haplotypes) say that haplotype 2 has "Assembly level: chromosome"?

Are sufficient details of methods and materials provided to allow replication by others?

Yes

Is the rationale for creating the dataset(s) clearly described?

Yes

Are the datasets clearly presented in a useable and accessible format?

Yes

Are the protocols appropriate and is the work technically sound?

Yes

Reviewer Expertise:

It is a standard Genome Note paper from the DToL project

I confirm that I have read this submission and believe that I have an appropriate level of expertise to confirm that it is of an acceptable scientific standard.
